# Infected or not: are PCR-positive oropharyngeal swabs indicative of low pathogenic influenza A virus infection in the respiratory tract of Mallard *Anas platyrhynchos*?

**DOI:** 10.1186/1297-9716-45-53

**Published:** 2014-05-14

**Authors:** Michelle Wille, Peter van Run, Jonas Waldenström, Thijs Kuiken

**Affiliations:** 1 Centre for Ecology and Evolution in Microbial Model Systems (EEMiS), Linnaeus University, SE - 391 82 Kalmar, Sweden; 2 Department of Viroscience, Erasmus Medical Centre, Dr. Molewaterplein 50, 3015 GE Rotterdam, The Netherlands

## Abstract

Detection of influenza virus in oropharyngeal swabs collected during wild bird surveillance is assumed to represent respiratory infection, although intestine is the main site of infection. We tested this assumption by histological examination of the respiratory tract of wild Mallards with virus-positive oropharyngeal swabs. Thirty-two of 125 Mallards tested had viral-RNA positive oropharyngeal swabs. The respiratory tracts of four Mallards with the most virus were examined in detail by immunohistochemistry. None had detectable virus antigen in the respiratory tract, suggesting it was not infected. An alternative explanation is that the oropharynx was contaminated with virus through feeding in surface water or through preening.

## Introduction, methods, and results

Influenza A viruses (IAV) have long been known to circulate in wild birds [1,2]. The main wild bird reservoir for low pathogenic avian influenza A (LPAI) viruses are Anseriformes (waterfowl) and some Charadriiformes (shorebirds, terns and gulls), with the highest prevalence detected in the Mallard *Anas platyrhynchos* in both North America and Europe [2]. LPAI viruses preferentially infect the epithelium of the lower gastrointestinal tract and are shed predominantly through the feces [3-5]. These viruses are thought to be transmitted mainly by fecal-oral route through bird-bird contact [1] and water-borne transmission [1,6]. An alternate infection route is cloacal drinking, i.e., the intake of fluids through the cloaca [7,8], which may play a role in the infection of the cloacal bursa of young birds [8]. Therefore, the recommended sample choice for LPAI virus surveillance is the cloacal swab. In contrast, highly pathogenic avian influenza (HPAI) H5N1 replicates preferentially in the respiratory tract, therefore, oropharyngeal swabs are often included in surveillance studies for HPAI viruses [9,10]. Both oropharyngeal swabs and cloacal swabs of birds may test positive for LPAI virus, and studies utilizing both sample types have described differences in detection rates, cycle threshold (Ct) values in the real-time PCR screen, and isolation success [11-14]. Further, the assumption that virus-positive oropharyngeal swabs represent respiratory tract infection has been introduced to the literature [11-15]. Support for this assumption is that receptors for LPAI viruses, α2,3 sialic acid receptors, are distributed not only in the gastrointestinal tract but also in the respiratory tract of Mallards [16,17]. Also, upon intra-tracheal inoculation of LPAI in Mallard, infection and replication of LPAI was detected along the length of the respiratory tract [18,19]. However, replication of LPAI virus in the respiratory tract has never been demonstrated in naturally infected birds [20]. The goal of this study was to determine whether positivity of oropharyngeal swabs reflects virus replication in the respiratory tract. Site of replication, and in turn, interpretation of oropharyngeal screening results, have implications in the methodology of continued and future surveillance schemes, and outcomes of epidemiological studies.

Wild, free-living Mallards were routinely sampled as part of a long-term surveillance program at Ottenby Bird Observatory, located in the southern Baltic Sea (56°13´ N 16°27´ E). We selected Mallard as it is an important maintenance host of LPAI virus, and as it is the most common reservoir host at the study site. The study was carried out in November (2012), one of the peak months of Mallard migration and influenza prevalence at the study site [21]. Across three sampling occasions, a total of 125 wild Mallards were caught in a baited trap and sampled; an oropharyngeal swab and either a cloacal or fresh fecal swab were collected from all individuals and placed in 1 mL of virus transport medium [21]. RNA was extracted within 1–4 h of collection and assayed using previous published methods [21]. Briefly, the sample was diluted 1:4 in PBS, and RNA extracted using the MagNA Pure 96 robot and the Viral NA Large Volume Kit (Roche, Mannheim, Germany). RNA was assayed for a short fragment of the matrix gene segment by RRT-PCR using the Light Cycler480 (Roche) and the One Step Real-Time PCR Kit (Qiagen, Hilden, Germany) and a value of less than 40 was considered positive. Thirty-two individuals were positive in the oropharyngeal swab (mean Ct-value 38.5), 28 were positive in the cloacal swab (mean Ct-value 35.3) and 12 were positive in both the oropharyngeal swab and cloacal swab simultaneously (Additional file 1). From these birds, five healthy individuals were selected, one as a negative control and four birds that had the lowest Ct-values in the oropharyngeal swab to increase the likelihood of detecting ongoing infection (Table 1). The RRT-PCR result of the cloacal swab did not play a role in animal selection. The selected birds were sacrificed using a CASH Poultry Killer (Accles & Shelvoke) within 8–10 h of original sample collection, following the results of the RRT-PCR screen. The remaining individuals were released.

**Table 1:** Detection of influenza A virus by RRT-PCR, virus isolation, and immunohistochemistry in Mallards from Ottenby

	**RRT-PCR (Ct value)**		**Virus isolation**		**Immunohistochemistry**		
**Individual**	**Oropharyngeal**	**Cloacal**	**Oropharyngeal**	**Cloacal**	**Respiratory**	**Gastrointestinal**	**Number of cells**
138909	Neg^a^	Neg	NA^b^	NA	Neg	Pos^c^	Cloacal bursa: 56 cells
138920	33.96	36.91	H4N6 E2^d^	Neg	Neg	Neg	
138964	36.14	33.7	H5N3 E2^d^	H5N3 E1^d^	Neg	Pos	Jejunum: 2 sections, 99 cells; Colon: 2 sections, 28 cells
138984	36.21	30.71	H4N6 E1^d^	Neg	Neg	Pos	Cloacal bursa: 2 sections, 62 cells
139211	33.59	29.34	Neg	Neg	Neg	Neg	

^a^Neg: not detected.

^b^NA: not analysed.

^c^Pos: detected.

^d^Passage number, where E1 is the first passage, E2 is the second passage, and E3 is the third passage.

RRT-PCR-positive samples were propagated in specific pathogen-free embryonated chicken eggs [21]. Up to three egg passages were performed, and allantoic fluid was assayed after each passage by hemagglutination. The hemagglutinin subtype of virus isolates was characterized using the hemagglutination inhibition test, and the neuraminidase subtype was determined using a PCR assay [22]. Influenza A virus from the oropharyngeal swabs of three of four birds selected were successfully propagated in eggs, and subtyped as H4N6 (*n* = 2) and H5N3 (*n* = 1). In one individual, the same virus subtype, H5N3, was isolated both from the oropharyngeal swab and the cloacal swab (Table 1). All sample screening and isolation were carried out at Linnaeus University, Sweden.

For virus antigen detection by immunohistochemistry, we collected all tissues from the respiratory tract of all five individuals; including trachea, bronchi, both left and right lung, and posterior air sacs. We also collected the entire head in order to section the nasal mucosa. As an internal, methodological control for immunohistochemical analysis, we also sampled and sectioned the gastrointestinal tract of all individuals: esophagus, proventriculus, duodenum, four seven-cm-long segments of jejunum at intervals of approximately seven centimeters, colon, cloaca, and cloacal bursa (if present). All tissues were fixed in 10% neutral-buffered formalin (Sigma) for 30 days prior to further processing.

After fixation, we mounted multiple sections from the length of each tissue to increase the chance of detecting infection, even if localized, as follows: four cross-sections of the nasal mucosa, 10 of the trachea, three of each bronchus, eight of each lung, seven of the esophagus, three of the proventriculus, one of the ventriculus, seven of the duodenum, seven of each jejunal segment (making 28 cross-sections of the jejunum in total), three of the colon, three of the cloacal bursa, and one of the cloaca. Following embedding in paraffin, 3-μm-thick sections were made and stained for the detection of IAV antigen as previously described [8,9,18]. Cells with distinct red staining in the nucleus were identified as sites of virus replication, and tissues were considered positive even if only one or a few positive cells were present. Duplicate sections were stained with hematoxylin and eosin (HE). Tissue sectioning and staining by HE and immunohistochemistry were conducted at Erasmus Medical Centre, The Netherlands.

**Figure 1:**
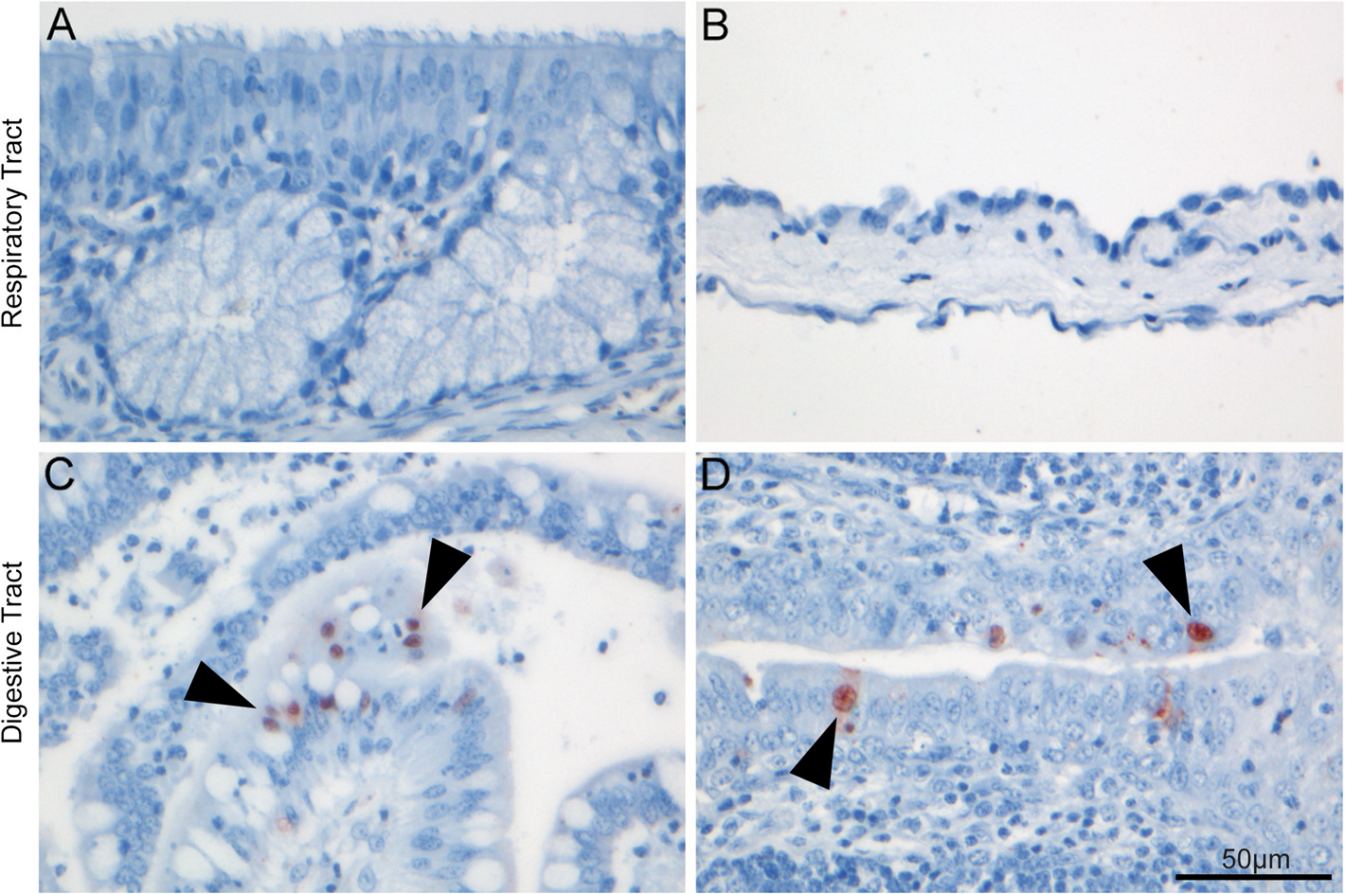
**Selected tissues of the respiratory and gastrointestinal tracts of Mallard following immunohistochemical staining to detect nucleoprotein of influenza A virus.** Tissues from the respiratory tract did not show virus antigen expression, such as **(A)** the respiratory epithelium of the nasal cavity and **(B)** air sac epithelium. In contrast, some tissues from the gastrointestinal tract such as **(C)** the epithelium lining the jejunal villi of the gastrointestinal tract, and **(D)** surface epithelium of the cloacal bursa did show virus antigen expression. Virus antigen expression is visible as diffuse to granular red staining, which is usually darker in the nucleus than in the cytoplasm. The tissues are counterstained blue with hematoxylin. Arrows have been included to illustrate positive cells. All images were captured at 400× magnification.

Despite testing multiple tissue sections along the length of the respiratory tract, none contained cells expressing IAV antigen (Table 1, Figure 1A-B). Three of 5 individuals were positive for IAV antigen in the gastrointestinal tract. The epithelium of the cloacal bursa (Figure 1D) was the only source of replicating virus in two individuals (138909, selected as the negative control; 138984), with 56 and 62 positive cells respectively. Multiple sections of the intestine were positive for antigens in individual 138964, where staining was located in the mucosal epithelium of the final segment of large intestine (2 sections, 99 positive cells) (Figure 1C), and the colon (2 sections, 28 cells). Despite having the lowest Ct-value, there was an absence of IAV antigen in the gastrointestinal tract of individual 139211. However, there was autolysis in many of the gastrointestinal sections, particularly affecting the tips of mucosal villi in this individual, and no positive cells were located in the lumen. There was no corresponding autolysis in any of the gastrointestinal segments of other individuals, or of respiratory sections from any individual. The absence of positive cells in individual 139211, despite being positive by RRT-PCR suggests this individual was not infected, but rather shedding detectable viral RNA. There was no inflammation or necrosis associated with the presence of influenza A virus antigen in the positive gastrointestinal sections, or respiratory sections, and none of the birds had gross lesions at dissection.

## Discussion

We were unable to demonstrate LPAI virus infection in the respiratory tract of any of the four Mallards in this study, despite choosing conditions and methods that maximized the chance of virus detection; the study was executed at a site with high IAV prevalence and during a period of peak prevalence; we chose individuals with the highest virus concentrations by RRT-PCR in the oropharyngeal swabs; and we performed detailed immunohistochemical analysis at all levels of the respiratory tract. These results indicate that these Mallards did not have LPAI virus infection in the respiratory tract despite testing positive in oropharyngeal swabs. Rather, we hypothesize that LPAI virus detection and isolation success in the oropharynx in these birds may be due to contamination of infectious particles from dabbling in virus-contaminated water or preening [23,24]. The involvement of virus-contaminated water is supported by the long persistence of LPAI virus in surface water and the importance of water-borne transmission in the epidemiology of avian influenza [25].

Immunohistochemistry is a sensitive method, and has been used to detect influenza A virus antigens in natural [8] and experimental LPAI [18,19] and HPAI infections [9,10] in waterfowl. The detection of IAV antigen in the lower gastrointestinal tract of three of five individuals shows that the immunohistochemical analysis on tissues from these birds functioned, despite a long fixation process. The detection of IAV antigen in the cloacal bursa of the bird with virus-negative cloacal swab by RRT-PCR indicates that the immunohistochemical analysis is sensitive. Further, previous studies have demonstrated the ability of this immunohistochemical analysis to detect IAV antigen in respiratory tract tissues of ducks with natural and experimental LPAI virus infection, and natural HPAI H5N1 virus infections [8-10,18]. Therefore, our immunohistochemical analysis was highly suited to detect LPAI virus infection in the respiratory tract of the Mallards in this study.

Low quantities of viral RNA may be detected by RRT-PCR in oropharyngeal/cloacal swabs for several days after abrogation of actual virus infection, based on virus isolation [26,27] and immunohistochemical detection in tissues [18,19]. However, we systematically selected individuals with the lowest Ct-values in the oropharyngeal swabs. The Ct-values we detected during the study period were comparable to values from our study site in previous years [11] and values from other studies of naturally infected waterfowl where respiratory tract infections have been suggested [11-15]. Additionally, infectious viruses were isolated from the oropharyngeal swabs of three individuals, across two different subtypes. The high rate of virus isolates obtained from RRT-PCR-positive swabs at our study site in this study period and previous years is probably attributable to the transport medium and the unbroken freeze chain, and supports the Ct cut-off value of 40 [11]. Together, it is unlikely that we were unable to include a single individual infected at the time of sampling using our study design.

One reason for the re-emergence of respiratory tract infection attribution, without evidence, in the literature is that these infections are present in experimental LPAIV infection [20] and in natural HPAIV infection. Respiratory tract infections in LPAIV experimental infections are likely related to the method of inoculation. Intraoral and intranasal inoculation involves flooding the oral and nasal cavities with inoculum, allowing aspiration of virus into the respiratory tract. Intratracheal inoculation purposely places the virus in the respiratory tract. These conditions do not reflect natural infection [20], and it has been demonstrated that different routes of inoculation do affect infectivity and viral shedding of Mallards [28]. For example, Daoust et al. [18] found virus antigen in the lungs and airsacs following intratracheal inoculation of an H2N3 virus, compared with a study by Franca et al. [19] where antigens were found in the trachea/larynx of a single individual one day post infection via the intranasal route.

In conclusion, we have demonstrated that IAV-positive oropharyngeal swabs obtained from wild migratory Mallards do not necessarily correspond to respiratory tract infection. The implication from this study is that Mallards that test positive for IAV only in the oropharyngeal swab should not automatically be categorized as being infected, especially if the Ct-values are high. Given that this study was performed on a limited number of Mallards infected with H4N6 or H5N3, further studies in both Mallards and other host species, naturally infected with different IAV subtypes, are needed to determine how to interpret IAV-positive oropharyngeal swabs from Mallards and other wild waterbird species sampled for influenza surveillance.

## Competing interests

The authors declare that they have no competing interests.

## Authors’ contributions

Conceived and designed the experiments: MW JW TK. Performed the experiments: MW, PvR. Analyzed the data: MW. Contributed reagents/materials/analysis tools: TK JW. Wrote the paper: MW JW TK. All authors read and approved the final manuscript.

## Supplementary Material

Additional file 1 **Ct values for all individuals that were positive.** Influenza A virus RNA was detected by RRT-PCR in oropharyngeal swabs, cloacal swabs, or both. Click here for file
