# Supplementary material for: Infected or not: are PCR-positive oropharyngeal swabs indicative of low pathogenic influenza A virus infection in the respiratory tract of Mallard Anas platyrhynchos?
Source: Vet Res. 2014 May 14;45(1):53. doi: 10.1186/1297-9716-45-53 (PMC4046040; doi:10.1186/1297-9716-45-53)
Supplement: Additional file 1 — Ct values for all individuals that were positive. Influenza A virus RNA was detected by RRT-PCR in oropharyngeal swabs, cloacal swabs, or both. [file 1297-9716-45-53-S1.docx]

| Ring Number | Sample ID | Ct value | | Date |
| --- | --- | --- | --- | --- |
|  |  | Oropharyngeal | Cloacal |  |
| 90A92361 | 138904 | 39.23 | 40 | 01-Nov-12 |
| 90A92362 | 138905 | 40 | 36.99 | 01-Nov-12 |
| 90A92363 | 138906 | 40 | 27.32 | 01-Nov-12 |
| 90A92364 | 138907 | 37.43 |  | 01-Nov-12 |
| 90A92365 | 138908 | 40 |  | 01-Nov-12 |
| 90A92367 | 138910 |  | 34.65 | 01-Nov-12 |
| 90A92368 | 138911 | 40 |  | 01-Nov-12 |
| 90A92370 | 138913 | 40 |  | 01-Nov-12 |
| 90A92360 | 138918 |  | 31.03 | 01-Nov-12 |
| 90A92358 | 138919 | 40 |  | 01-Nov-12 |
| 90A92359^a^ | 138920 | 33.96 | 36.91 | 01-Nov-12 |
| 90A92366^a,b^ | 138909 |  |  | 01-Nov-12 |
| 90A92372 | 138938 | 38.86 |  | 02-Nov-12 |
| 90A92373 | 138939 | 40 |  | 02-Nov-12 |
| 90A92374 | 138940 | 38.72 |  | 02-Nov-12 |
| 90A92375 | 138941 |  | 38.18 | 02-Nov-12 |
| 90A92376 | 138942 |  | 35.45 | 02-Nov-12 |
| 90A92377 | 138943 |  | 40 | 02-Nov-12 |
| 90A92379 | 138945 |  | 31.06 | 02-Nov-12 |
| 90A92381 | 138947 | 40 | 34.51 | 02-Nov-12 |
| 90A92382 | 138948 | 38.55 |  | 02-Nov-12 |
| 90A92383 | 138949 | 38.52 | 40 | 02-Nov-12 |
| 90A92385 | 138951 | 40 |  | 02-Nov-12 |
| 90A92386 | 138952 | 39.41 |  | 02-Nov-12 |
| 90A92389 | 138955 | 40 |  | 02-Nov-12 |
| 90A92393 | 138959 | 38.25 |  | 02-Nov-12 |
| 90A92395 | 138961 | 40 |  | 02-Nov-12 |
| 90A92396 | 138962 | 38.41 |  | 02-Nov-12 |
| 90A92398^a^ | 138964 | 36.14 | 33.7 | 02-Nov-12 |
| 90A92320 | 138973 |  | 40 | 02-Nov-12 |
| 90A88456 | 138980 |  | 37.81 | 02-Nov-12 |
| 90A92316 | 138981 |  | 37.33 | 02-Nov-12 |
| 90A85840 | 138982 | 39.46 |  | 02-Nov-12 |
| 90A87023 | 138983 | 38.99 |  | 02-Nov-12 |
| 90A92156^a^ | 138984 | 36.21 | 30.71 | 02-Nov-12 |
| 90A92152 | 138985 |  | 37.99 | 02-Nov-12 |
| 90A92162 | 138986 | 40 | 35.65 | 02-Nov-12 |
| 90A92135 | 138991 |  | 33.99 | 02-Nov-12 |
| 90A92434 | 139188 | 37.82 |  | 10-Nov-12 |
| 90A92437 | 139191 | 37.59 |  | 10-Nov-12 |
| 90A92364 | 139198 |  | 35.07 | 10-Nov-12 |
| 90A92316 | 139201 |  | 34.78 | 10-Nov-12 |
| 90A92372 | 139202 | 37.67 | 31.24 | 10-Nov-12 |
| 90A85596 | 139205 | 37.01 |  | 10-Nov-12 |
| 90A92320^a^ | 139211 | 33.59 | 29.34 | 10-Nov-12 |
| 90A92317 | 139212 |  | 34.92 | 10-Nov-12 |
| 90A92375 | 139214 |  | 36.93 | 10-Nov-12 |
| 90A92339 | 139223 |  | 37.73 | 10-Nov-12 |
| 90A92373 | 139227 | 37.03 | 35.93 | 10-Nov-12 |

^a^ individuals that were sacrificed in this study.

^b^ negative control.
